# Reflections of an intensivist in 2050: three decades of clinical practice, research, and human connection

**DOI:** 10.1186/s13054-023-04674-5

**Published:** 2023-10-09

**Authors:** Maurizio Cecconi

**Affiliations:** 1 Biomedical Sciences Department, Humanitas University, Milan, Italy; 2 Department of Anaesthesia and Intensive Care, IRCCS Humanitas Research Hospital, Milan, Italy

I woke up after a vivid dream this morning: It is a crisp day in 2050, and many memories whirl through my mind, evoking the nostalgia of over three transformative decades in intensive care.

The year 2020 seems a lifetime ago. The world faced a pandemic that tested our preparedness, strength, resilience, and dedication to our patients. But as I look back, it is evident that the fundamental principles that guided us back then remain as crucial today as they were 30 years ago [1, 2].

Initially, intensive care was a microcosm of the grand dance between human physiology, technology, and humanity. As intensivists, we prided ourselves on understanding the most challenging questions of the human body, the delicate interplay of organs, and the profound resilience of the human spirit.

But in the era that followed, a new wave of transformation swept our world: the age of phenotyping, adaptive research, and artificial intelligence. This was no ordinary change. It was a massive shift in how we approached our patients.

We began to delve deeper into the subsets of populations, unravelling the unique intricacies of individual patient physiology. Rather than treating every patient with a one-size-fits-all approach, we were now guided by precise, brilliant, adaptive, randomised studies tailored to small, homogenous populations. Artificial intelligence amplified our intuition, enriching our insights with vast troves of data, but in an intimately personalised way.

As we made these strides in understanding the human body at an unprecedented level, something remarkable happened: Our relationships with our patients and their families deepened. It was as if the more we understood the individuality of human physiology, the more we came to cherish the irreplaceable human soul.

By 2050, walking into an ICU feels like stepping into the future. Large, interactive screens replace the more minor, cluttered monitors of yesterday. Automated fluid challenge systems allow us to optimise patient haemodynamics with unprecedented precision. When a trend or anomaly needs attention, an orange lamp blinks.

Consider Sarah, for instance, 65 years old, admitted with septic shock. An orange lamp blinks, drawing my attention to a Pseudomonas infection revealed by her bloodwork. I consult the avatar of an infectious disease specialist, and together, we tailor an antibiotic regimen specifically for Sarah. This technology frees me to spend precious moments with Sarah's family, discussing her condition, calming their fears, and answering their questions in a way I could not before.

Specialists no longer need to be physically present; avatars appear on screens, providing expert consultations in real time. This technology does not replace us; it empowers us. Now, we have more time to spend with patients and their families, discussing the nuances of care and making shared decisions, Table 1.

**Table 1:** ICU evolution: a comparative look

Aspect	ICU in 2020	ICU in 2050
Monitoring	Standard monitors	Large, interactive screens
Consultation	In-person specialists—telemedicine	Avatars of specialists
Treatment	One-size-fits-all	Personalised, adaptive therapies
Family interaction	Limited due to time constraints	Ample time for meaningful conversations

It would be easy to assume that with technological advances, the essence of our role would diminish and that machines and algorithms would replace the need for human connection. But the opposite occurred. Our expertise and emerging technology brought us closer to our patients. We became their guides, navigating them through a world where science met souls, explaining the nuances, reassuring their fears, and sharing their joys and sorrows.

Behind every ventilator beep, every AI-generated alert was in the hands of our incredible team.

I wish we had taken care better of our teams in 2020. Many suffered from moral injury, burnout, and disengagement. Some left the profession. Luckily, after some rocky years, doctors and nurses got their enthusiasm back, and I could not think of a more rewarding place to work in an ICU Team [3].

Intensive care teams have become even more important: our doctors, who, with every passing year, combined their vast knowledge with compassion; our nurses, whose attentive care was the bedrock of our units; our physiotherapists, who breathed life back into weary limbs and spirits; and any other healthcare worker with their unique contribution to our teams. This was always a team effort, and our collective strength was the magic that wove science and humanity together [4].

The simple, heartfelt conversations with a patient's family were some of the most profound moments in the labyrinth of tubes, wires, and machines. The late-night discussions, where we reassured, educated, and sometimes grieved together, stand out as some of our most important contributions to healing.

Today, I want to speak directly to the young intensivists just beginning their journeys.

- Stay Curious: Technology will keep evolving, but human physiology and care fundamentals remain constant.
- Embrace but Don't Rely on Technology: Use it to aid your expertise, not replace it.
- Connect: Spend extra time connecting with patients and their families. Human interaction is irreplaceable.
- Remember, while machines can predict, analyse, and compute, they cannot comfort a grieving mother, share a joke to lighten a patient's heart, or offer a hand of reassurance when fear clouds the eyes of a family member.

As I prepare to retire, my heart swells with gratitude for the journey and the countless souls I’ve had the privilege to serve alongside and care for. The future of intensive care medicine is bright, illuminated by the fusion of ground-breaking technology and the timeless human spirit. To the next generation of intensivists: Nurture your passion for intensive care. Merge science with soul. You embrace the most rewarding journey you could have imagined when you started.

## Data Availability

Not applicable.
